# Application of metagenomic next-generation sequencing in the detection of pathogens in bronchoalveolar lavage fluid of infants with severe pneumonia after congenital heart surgery

**DOI:** 10.3389/fmicb.2022.954538

**Published:** 2022-08-05

**Authors:** Yi-Rong Zheng, Shi-Hao Lin, Yu-Kun Chen, Hua Cao, Qiang Chen

**Affiliations:** ^1^Department of Cardiac Surgery, Fujian Branch of Shanghai Children’s Medical Center, Fuzhou, China; ^2^Fujian Children’s Hospital, Fuzhou, China; ^3^Fujian Maternity and Child Health Hospital, College of Clinical Medicine for Obstetrics & Gynecology and Pediatrics, Fujian Medical University, Fuzhou, China; ^4^Fujian Key Laboratory of Women and Children’s Critical Diseases Research, Fujian Maternity and Child Health Hospital, Fuzhou, China

**Keywords:** metagenomics next-generation sequencing, bronchoalveolar lavage fluid, pneumonia, infection, congenital heart surgery

## Abstract

**Background:**

Metagenomic next-generation sequencing (mNGS) has become a valuable diagnostic tool in clinical etiology detection due to its rapidity, accuracy, and high throughput. However, the role of this technology in the diagnosis and treatment of infants with severe pneumonia after congenital heart surgery is still unclear.

**Methods:**

We conducted a retrospective cohort study of infants with severe pneumonia after congenital heart surgery. Samples were collected from infants in the hospital’s cardiac intensive care unit between January 2010 and January 2022. The conventional microbiological test (CMT) group consisted of patients who underwent routine microbiological examination, and the infants’ bronchoalveolar lavage fluid was examined. The mNGS group consisted of patients who underwent mNGS and routine microbiological examinations.

**Results:**

The overall positive rate of mNGS was significantly higher than that of CMT (88.4 vs. 62.5%, *P* = 0.009). After receipt of the microbiological results, 30/43 (70%) patients in the mNGS group had a change in antibiotic use compared with 14/40 (35%) in the CMT group (*P* = 0.002). Subsequently, after adjusting the treatment plan according to the microbiological test results, the number of people with improved pulmonary infection in the mNGS group was significantly higher than that in the CMT group (63 vs. 28%, *P* < 0.05). In addition, the duration of invasive ventilation, length of CICU stay and total hospital length of stay in the mNGS group were significantly lower than those in the CMT group (*P* < 0.05).

**Conclusion:**

mNGS is a valuable tool to determine the etiology of infants with severe pneumonia after congenital heart disease surgery. It can significantly improve the sensitivity of pathogen detection, which can help determine appropriate antimicrobial drugs, improve the diagnostic accuracy of the disease, and improve outcomes.

## Introduction

Pneumonia is one of the main causes of postoperative morbidity and mortality in infants with congenital heart disease (CHD). They are at high risk of infection for a variety of reasons including malnutrition, pulmonary congestion, ischemia–reperfusion injury after cardiopulmonary bypass, and congenital immunodeficiencies such as DiGeorge syndrome (Murni et al., 2017). Early and appropriate antimicrobial therapy against the pathogen is a major component of pneumonia treatment while preventing the overuse of antibiotics and the emergence of resistance (Bradley et al., 2011; Vaughn et al., 2019). Accurate pathogen detection is a prerequisite for precise antimicrobial treatment. Infants with severe pneumonia after congenital heart surgery (CHS) often require tracheal intubation, and bronchoalveolar lavage fluid (BALF) has become a relatively easy-to-obtain clinical specimen with high diagnostic value. Conventional microbiological tests (CMTs) of BALF, such as bacterial and fungal smears and cultures, do not meet the diagnostic needs of critically ill patients due to long detection cycles and low sensitivity (Jain et al., 2015; Zhou et al., 2019). Culture-independent assays (serological assays and nucleic acid amplification assays) have been shown to be effective methods for determining etiology. However, polymerase chain reaction kits are not comprehensive in terms of breadth and resolution due to their limited detection range. Recently, metagenomic next-generation sequencing (mNGS) has been widely used in the diagnosis and treatment of infectious diseases, and studies have found that it is helpful to improve prognosis and reduce mortality (Miao et al., 2018; Xie et al., 2019; Cai et al., 2020; Qian et al., 2020; van Boheemen et al., 2020). Furthermore, an increasing number of rare pathogens have been detected by mNGS methods, providing strong evidence for the diagnosis and treatment of some difficult and critically ill patients (Zhang et al., 2019; Wang et al., 2020). However, there are no application studies of mNGS in cardiac intensive care unit (CICU) patients with severe pneumonia. This study summarizes clinical data through retrospective analysis and explores the clinical value of mNGS in detecting pathogens in BALF of patients with severe pneumonia after CHS.

## Materials and methods

### Population and study criteria

Infants with severe pneumonia after CHD admitted to the CICU of Fujian Maternal and Child Health Hospital and Fujian Children’s Hospital from January 2020 to January 2022 were retrospectively analyzed. The inclusion criteria were as follows: (1) The diagnosis of severe pneumonia was based on the criteria established by the Paediatric Infectious Diseases Society and the Infectious Diseases Society of America; (2) BALF was obtained via bedside bronchoscopy; (3) BALF was sent for at least 1 mNGS examination; (4) BALF was simultaneously detected by the CMT method, including at least bacterial and fungal smears and cultures; (5) Routine blood and inflammatory markers, including CRP, PCT, (1,3)-β-D-glucan and galactomannan antigens, were detected before and 24 h after BALF collection. The exclusion criteria were as follows: (1) BALF samples that failed mNGS quality control, e.g., sequences of human origin exceeded 99%; (2) sample leakage and contamination; and (3) incomplete clinical history. Patients were included in the mNGS group when informed consent was provided for testing while those who were not tested by mNGS were grouped into the CMT group.

### Sample processing and conventional microbiological test

Bedside bronchoscopy was performed by the attending physicians after the patient’s written informed consent. Physicians followed standard procedures to collect 3–5 mL of BALF samples from the infant. Each sample was placed in a sterile sputum container and then separately sent to the microbiology laboratory for routine examination such as bacterial and fungal smears and cultures, acid-fast staining, and polymerase chain reaction testing of specimens from patients with suspected viral infection.

### Metagenomic next-generation sequencing procedure

Samples of 3–5 mL were collected from infants following standard procedures. A 1.5 mL microcentrifuge tube containing 0.5 mL BALF sample and 1 g 0.5 mm glass bead was connected to the horizontal platform of the vortex mixer. Then, the mixture was stirred vigorously at 3,000 RPM for approximately 30 min. The 0.3 mL sample was transferred into a new 1.5 mL microcentrifuge tube using a TIANamp microtubule and DNA extraction DNA kit (DP316, Tiangen Biotechnology) according to the manufacturer’s recommendations. After that, DNA libraries were constructed by the methods of DNA fragmentation, end repair, adapter ligation, and PCR amplification. An Agilent 2100 was used for DNA library quality control. Qualified libraries were sequenced by the BGISEQ-50 platform (Fang et al., 2018).

### Bioinformatics analysis

**FIGURE 1:**
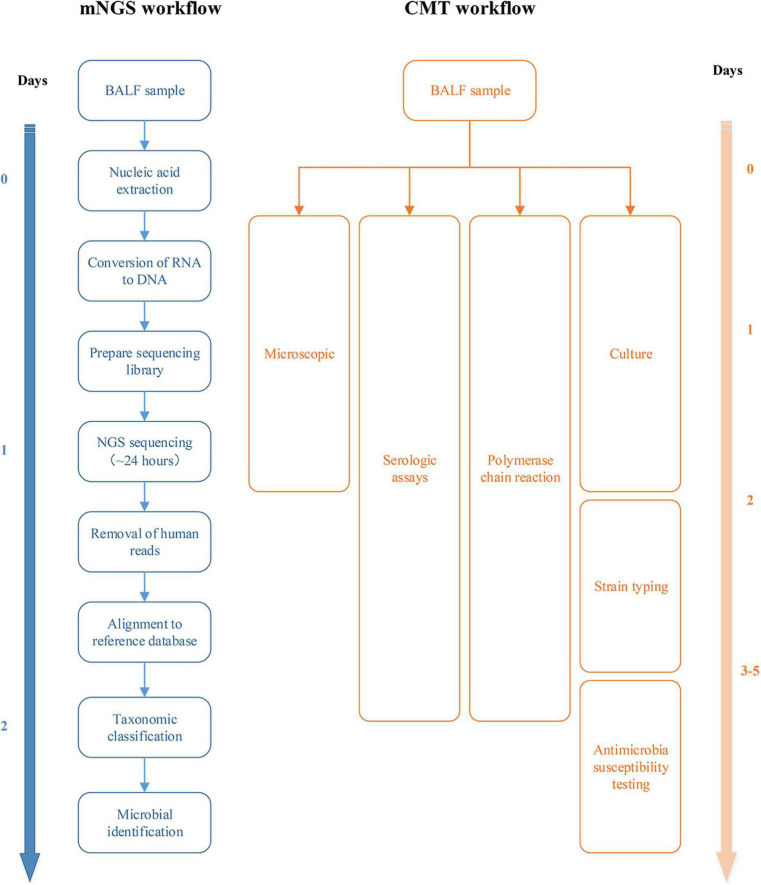
Workflow and timeline for metagenomic next-generation sequencing (mNGS) and conventional microbiological test (CMT).

Quality control of raw sequencing data, including removal of low-quality, low-complexity, short reads (<35 bp) and adapter trimming followed by alignment of the human reference genome (hg38) using Burrows–Wheeler alignment, was performed and reads obtained in the human genome were discarded. Microbial classification was performed by mapping the remaining sequenced fragments to a reference microbial database consisting of the genomes of archaea, bacteria, fungi, protozoa, viruses, and parasites from the NCBI Genome Database (Benson et al., 2013). The number of unique alignment reads was calculated and standardized to obtain the number of reads stringently mapped to pathogen species and the number of reads stringently mapped to pathogen genera. The workflow and timeline for mNGS and CMT are shown in Figure 1.

### Statistical analysis

Data were analyzed using SPSS software version 25.0 for Windows (IBM SPSS Inc., Chicago, IL, United States). Independent continuous variables were presented as the mean ± standard deviation (SD) and were analyzed by *t*-tests. Counts and percentages describe the enumeration data. Means were compared using Student’s *t*-test, and Fisher’s exact test was used for categorical data. The Mann–Whitney *U* test was applied for non-normally distributed data. A two-sided *P*-value of < 0.05 was regarded as statistically significant.

## Results

### Demographic characteristics

A total of 88 infants with ARDS caused by severe pneumonia were screened in this study, and 5 patients were excluded according to the exclusion criteria. Among them, 2 patients had incomplete clinical history data, 2 failed mNGS quality control, and 1 had BALF sample contamination. Forty-three infants were included in the NGS group, and 40 were included in the CMT group. The clinical characteristics of the NGS group and CMT group, including demographic characteristics, incidence of pulmonary hypertension and preoperative respiratory infection, type of CHD, surgical information, postoperative bronchoscopy time, laboratory test results, ventilator parameters, etc., are shown in Tables 1, 2, with no statistically significant differences between the two groups (*P* > 0.05). In this study, all patients were treated with empirical antibiotics. Bronchoscopy was used for diagnosis or treatment, and BALF examination was performed. There was no statistically significant difference in the time of bronchoscopy after surgery, but the time of sample submission to results feedback in the mNGS group was significantly shorter than that in the CMT group, and the difference was statistically significant (25.6 ± 3.5 h vs. 78.5 ± 5.5 h, *P* < 0.05). All enrolled patients received only one mNGS test. There were no statistically significant differences in laboratory data or ventilator parameters between the two groups during the study period (Table 2).

**TABLE 1:** Clinical characteristics of included patients^a^.

Characteristics	NGS (*n* = 43)	CMT (*n* = 40)	*P*-value
Sex (Male/Female)	23/20	18/22	0.512
Age (months; mean ± SD)	2.8 ± 1.1	3.0 ± 0.9	0.175
Weight (kg; mean ± SD)	5.5 ± 0.6	5.3 ± 0.9	0.436
Pulmonary hypertension, *n* (%)	31 (72)	26 (65)	0.636
Preoperative respiratory infection, *n* (%)	25 (58)	19 (48)	0.383
**Congenital heart disease**			
VSD	9	9	
VSD + ASD	9	8	
TAPVC	7	6	1.000
COA	5	4	
TOF	4	4	
DORV	3	3	
IAA	2	3	
PDA	2	2	
TGA	2	1	
CPB time (minutes; mean ± SD)	115.7 ± 25.8	116.1 ± 29.2	0.960
Aortic cross-clamp time (minutes; mean ± SD)	57.1 ± 19.1	54.4 ± 21.0	0.536
Postoperative bronchoscopy time (days; mean ± SD)	3.8 ± 0.9	4.1 ± 1.1	0.075
Time of sample submission to results feedback (hours; mean ± SD)	30.6 ± 3.5	78.5 ± 5.5	< 0.001

mNGS, metagenomic next-generation metagenomic sequencing; CMT, conventional microbiological test; VSD, ventricular septal defect; ASD, atrial septal defect; TAPVC, total anomalous pulmonary venous connection; COA, coarctation of the aorta; TOF, tetralogy of Fallot; DORV, double outlet of right ventricle; IAA, interrupted aortic arch; PDA, patent ductus arteriosus; TGA, transposition of the great arteries; CPB, cardiopulmonary bypass.

^a^Data reported as number and percentage or mean ± standard deviation.

**TABLE 2:** Laboratory data and ventilator parameters during the study period^a^.

Variable	mNGS (*n* = 43)	CMT (*n* = 40)	*P*-value
WBC (10^9^/L)	11.5 (6.9, 19.4)	13.3 (7.3, 17.5)	0.185
Percentage of neutrophils (%)	70.5 (63.1, 80.7)	63.6 (57.3, 75.5)	0.275
CRP (mg/L)	21.5 (15.1, 47.8)	25.3 (18.3, 51.2)	0.776
PCT (ng/mL)	7.5 (2.7, 17.6)	8.1 (3.4, 18.3)	0.605
Scr (mmol/L)	76 (64, 203)	97 (67, 134)	0.512
ALT (IU/L)	31 (25, 51)	27 (25, 55)	0.602
Lactate (mmol/L)	1.4 (1.1, 2.9)	1.7 (1.0, 2.5)	0.761
NT-proBNP (pg/mL)	882 (575, 3563)	905 (477, 3866)	0.488
FiO_2_	0.8 (0.6, 1.0)	0.6 (0.5, 0.8)	0.932
PEEP	6 (4, 10)	5 (4, 8)	0.275
MAP	7 (5, 15)	8 (6, 15)	0.853
OI at inclusion	9 (6, 15)	8 (6, 17)	0.686

mNGS, metagenomic next-generation sequencing; CMT, conventional microbiological test; WBC, White blood cell; CRP, C-reactive protein; PCT, Procalcitonin; Scr, Serum creatinine; ALT, Alanine aminotransferase; NT-proBNP, N-terminal Pro-Brain Natriuretic Peptide; FiO_2_, Fraction of inspiration O_2_; PEEP, positive end-expiratory pressure; MAP, mean airway pressure; OI, Oxygenation Index.

The hospital reference ranges: WBC (10^9^/L): 4.5–9.5; percentage of neutrophils (%): 45–75, CRP (mg/L): 0–10; PCT (ng/mL): < 0.05, Scr (mmol/L):13–33, ALT (IU/L): 20–40; lactate (mmol/l):0.1–1; NT-proBNP (pg/mL): 0–125.

There were no differences in laboratory examination, ventilator parameters before treatment between two groups (*P* > 0.05).

*^a^*The measured data of patients’ physiological indicators in the above table were shown by median (interquartile range).

### Comparison of metagenomic next-generation sequencing and conventional microbiological test pathogen detection methods

**FIGURE 2:**
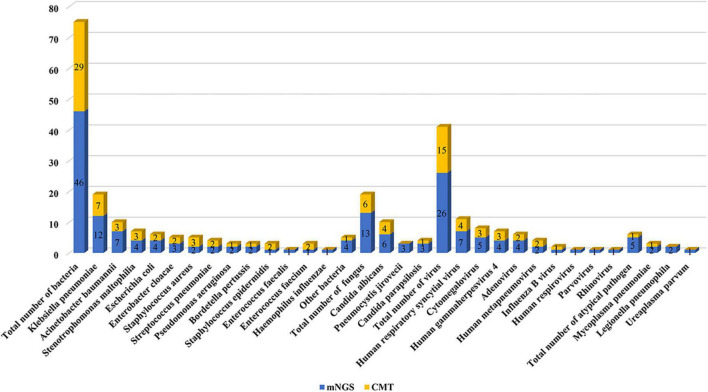
Pathogen distribution detected by metagenomic next-generation sequencing (mNGS) and conventional microbiological test (CMT).

**FIGURE 3:**
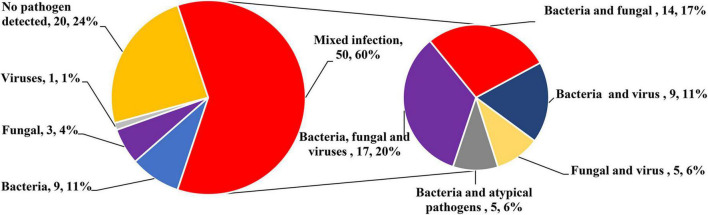
Percentage of patients with mixed infection.

Figure 2 lists the distribution of all detected major bacteria, fungi, viruses, and other atypical pathogens. There was no significant difference in the proportion of each species between the two groups. In terms of bacteria, the mNGS group had a significantly higher detection rate than the CMT group (93 vs. 45%; *P* < 0.001). The most common pathogens in both groups were *Klebsiella pneumoniae* (mNGS vs. CMT: 26 vs. 24%; *P* = 0.732), followed by *Acinetobacter baumannii* (9 vs. 10%; *P* = 0.393), and *Stenotrophomonas maltophilia* (9 vs. 10%; *P* = 1.000). In terms of fungi, the detection rate of the mNGS group was also higher than that of the CMT group (30 vs. 15%; *P* = 0.121), but the difference was not statistically significant. Fungus was detected in 13 patients in the mNGS group, with the most common fungi being *Candida albicans* in 6 cases, *Pneumocystis jirovecii* in 4 cases, and *Candida parapsilosis* in 3 cases. The most common fungi in the CMT group were *Candida albicans* in 3 cases and *Candida parapsilosis* in 1 case. In terms of viruses, there was no significant difference in the detection rate between the mNGS group and the CMT group (53 vs. 33%; *P* = 0.076). Viruses were detected in 23 patients in the mNGS group, and the most frequently detected viruses were respiratory syncytial virus in 7 cases, cytomegalovirus in 5 cases, and Epstein–Barr virus in 4 cases. In the CMT group, only 25 patients with suspected viral infection were tested for viruses. A total of 13 patients tested positive for the virus, of which the top three viruses were respiratory syncytial virus (4 cases), cytomegalovirus (3 cases), and Epstein–Barr virus (3 cases). In terms of atypical pathogens, there was no significant difference in the detection rate between the mNGS group and the CMT group (12 vs. 3%; *P* = 0.203). mNGS detected 1 case of *Mycoplasma pneumoniae*, 2 cases of *Legionella pneumophila*, and 1 case of *Ureaplasma urealyticum*, while only 1 case of *Mycoplasma pneumoniae* was detected by CMT. The overall positive rate of mNGS (38/43, 88.4%) was significantly higher than that of CMT (25/40, 62.5%, *P* = 0.009). In the mNGS group, 14 cases (32.6%) were copositive for both methods (CMT and mNGS). In addition, 60% of infants in both groups had mixed infections, and multiple microbial infections were found more frequently in the mNGS group than in the CMT group (88% vs. 30%, *P* < 0.001). The percentage of coinfection with various pathogens in the two groups is shown in Figure 3. Among them, bacterial-fungal-viral coinfection was the most common (17 and 20%), followed by bacterial-fungal (14 and 17%), bacterial-viral (9 and 11%), fungal-viral (5 and 6%), and bacterial-atypical pathogens (5 and 6%) in the mNGS and CMT groups, respectively.

### Treatment and prognosis

**FIGURE 4:**
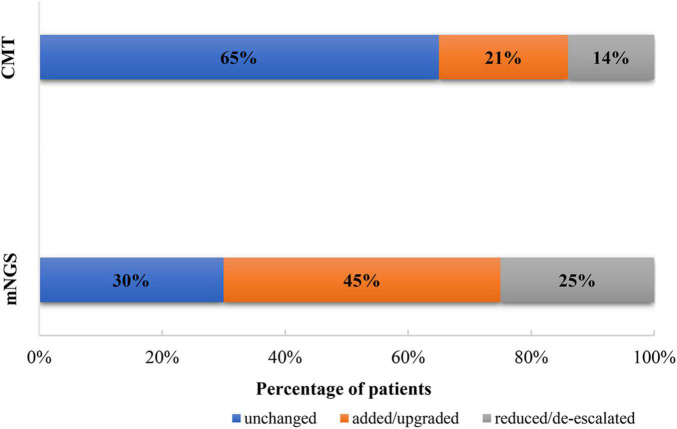
Adjustment of antibiotics based on metagenomic next-generation sequencing (mNGS) and conventional microbiological test (CMT) results.

After obtaining the mNGS results, we adjusted the antibiotic regimen according to the following principles: (1) The original anti-infection regimen was maintained. (2) The anti-infection regimen was changed according to the clinical characteristics of the patients and the detection results of pathogenic bacteria: a. When there was a new infectious pathogen, we added or changed antibiotic therapy. b. When there were no new infections and the disease was stable, we reduced or de-escalated the antibiotics. In this study, 30/43 (70%) patients in the mNGS group had antimicrobial adjustments: in 45% of cases, new antibiotics were added or the current antibiotics were upgraded and in 25% of cases, the current antibiotics were reduced or de-escalated. In the CMT group, 14/40 (35%) had antibiotic adjustments: in 21% of cases, new antibiotics were added or the current antibiotics were upgraded and in 14% of cases, the current antibiotics were reduced or de-escalated (Figure 4).

After receiving microbiological test results and adjusting for antibiotics, pulmonary infection improved in 27/43 patients (63%) in the mNGS group compared with 11/40 patients (28%) in the CMT group in the ensuing 7 days (*P* = 0.032) (Table 3). For example, in an infant after CHS, computed tomography showed diffuse changes in both lungs, and mNGS detected *Pneumocystis jirovecii*. According to the patient’s clinical manifestations, the clinician prescribed caspofungin and compound sulfamethoxazole tablets for the treatment of pneumonia. After antimicrobial treatment, the condition of the infant improved quickly, and he was cured and discharged. The time required for mechanical ventilation was shorter in the mNGS group than in the CMT group (3.6 ± 1.9 vs. 4.6 ± 2.2 days; *P* = 0.030). In addition, the duration of ICU stay and total hospital length of stay were shorter in the mNGS group than in the CMT group (5.8 ± 3.3 days vs. 7.5 ± 3.0 days, *P* = 0.010 and 15.3 ± 4.1 days vs. 17.1 ± 3.5 days, *P* = 0.033, respectively). Finally, in-hospital death occurred in 1 patient in the mNGS group and 3 patients in the CMT group after the diagnosis of severe pneumonia (*P* = 0.348).

**TABLE 3:** Comparison of outcomes between mNGS and CMT groups^a^.

Variable	mNGS (*n* = 43)	CMT (*n* = 40)	*P*-value
Pulmonary infections improved, *n* (%)	27 (63)	11 (28)	**0.002**
In-hospital mortality, *n* (%)	1 (2)	3 (8)	0.348
ECMO, *n* (%)	1 (2)	2 (5)	0.607
CRRT, *n* (%)	5 (12)	3 (8)	0.713
Duration of invasive ventilation (days; mean ± SD)	3.6 ± 1.9	4.6 ± 2.2	**0.030**
CICU length of stay (days; mean ± SD)	5.8 ± 3.3	7.5 ± 3.0	**0.010**
Total hospital length of stay (days; mean ± SD)	15.3 ± 4.1	17.1 ± 3.5	**0.033**

mNGS, metagenomic next-generation metagenomic sequencing; CMT, conventional microbiological test; ECMO, extracorporeal membrane oxygenation; CRRT, continuous renal replacement therapy; CICU, cardiac intensive care unit.

^a^Data reported as number and percentage or mean ± standard deviation. Values in bold indicated statistical differences between the two groups.

## Discussion

Pulmonary infection is a common infectious disease after CHS, especially for infants with high morbidity and high mortality. The main reasons for the poor efficacy of empirical treatment of pneumonia are uncertain pathogens and mixed infections. Rapid and accurate detection of the pathogen is critical for the treatment and prognosis of patients with pulmonary infections. mNGS is a highly sensitive assay for the simultaneous detection of numerous pathogens in clinical samples (Kawada et al., 2016). In this study, mNGS and CMTs were performed on the BALF of 83 infants with severe pneumonia after CHS. The results showed that mNGS was significantly more sensitive than CMT for BALF detection, shortened the time to diagnose pathogens, and promoted the target. There are considerable advantages of mNGS over CMT in terms of determining appropriate antibiotic therapies and improving patient outcomes.

The sensitivity, timeliness, and specificity of CMT techniques are not ideal, and they are easily affected by antimicrobial drugs (Nath, 2015). Due to factors such as the limitation of culture conditions, many pathogens cannot be cultured under existing laboratory conditions. Furthermore, pulmonary infection after CHS is often mixed infection, making it more difficult to culture. mNGS can overcome the limitations of CMT and perform broad-spectrum detection of pathogens, including bacteria, viruses, fungi and parasites. This study found that mNGS is more time-sensitive than the CMT method for detecting pathogenic microorganisms. It takes only 1–2 days on average from sending samples to receiving reports, while conventional culture takes at least 3–5 days. In addition, for bacteria, fungi, and viruses, the positive rates of the mNGS group were significantly higher than those of the CMT group. Relevant studies have found that mNGS technology can identify pathogens in patients with severe pneumonia early and guide the use of antibiotics, thereby significantly reducing the mortality rate of children with severe pneumonia (Xie et al., 2019). At present, mNGS has been widely used in the detection of pathogenic microorganisms in body fluids, such as sputum and BALF, in respiratory infectious diseases and has good diagnostic value (Li et al., 2018).

Severe pneumonia after CHS often requires mechanical ventilation, samples are easy to obtain by bronchoalveolar lavage, and it is well tolerated. mNGS is suitable for the detection of pathogens that other conventional techniques cannot identify and in cases where patients do not respond to standardized antimicrobial therapy. Overuse of antibiotics can lead to drug resistance and wasted health care resources (Ramirez et al., 2020). Because mNGS can provide faster and more pathogenic bacteria detection information, it can help clinicians adjust antibiotics in a timelier manner, including addition and subtraction of antibiotics, upgrading and escalation of antibiotics, and initiation of targeted anti-infection therapy. In this study, the number of antibiotic adjustments in the mNGS group was significantly higher than that in the CMT group (70 vs. 35%, *P* < 0.05), and the proportion of pulmonary infections that improved after adjustment was also significantly higher (63 vs. 28%, *P* < 0.05). Apparently, patients in the mNGS group received more targeted treatment after obtaining the aetiological report. However, it should be noted that mNGS results should be combined with the relative abundance of the assay and epidemiological and clinical characteristics to identify the causative microorganism.

We found that the proportion of mixed infections after CHD was high (60%). In addition, the detection rate of mNGS mixed infection (48.9%) was significantly higher than that of conventional pathogen detection methods (4.3%), suggesting that mNGS has a strong advantage in the diagnosis of mixed infectious pathogenic microorganisms (Parize et al., 2017). Previous studies have reported that identification of unsuspected rare pathogens is a major advantage of mNGS (Gu et al., 2020). In our study, mNGS detected specific pathogens that are difficult to culture in children with severe pneumonia after CHS, including *Bordetella pertussis, Pneumocystis jirovecii*, and cytomegalovirus. Because these pathogens often require special growth conditions, they are difficult to identify by traditional methods. Fortunately, the advantage of mNGS is that it can sequence hundreds of thousands to millions of DNA molecules simultaneously and enables comprehensive analysis of the transcriptome and genome of the same species. After that, it compares and intelligently analyzes the detection results with the reference pathogenic microorganism sequences in the database to calculate the sequence number of various pathogenic microorganisms and avoid missed detection (Barlow et al., 2007; Pan et al., 2008). For example, in this study, a patient with a double outlet right ventricle developed severe pneumonia after surgery. The white blood cells in the routine blood tests increased to 38 × 10^9^/L at one time, and the effect was poor after conventional antimicrobial treatment. The mNGS results identified *Bordetella pertussis*. The infection improved quickly after adjusting to azithromycin antimicrobial treatment.

Previous studies have suggested that treatment decisions based on mNGS results may have better clinical outcomes than routine examinations in intensive care unit patients with severe pneumonia (Xie et al., 2019). We also found that mNGS technology can significantly shorten the CICU stay and the duration of mechanical ventilation in infants with severe pneumonia after CHS. From the perspective of economics and clinical prognosis, children with CHD complicated with severe pneumonia are more suitable for the early clinical application of mNGS technology to assist in clinical diagnosis and drug decision-making. Although the number of in-hospital deaths in the mNGS group was lower than that in the CMT group, the difference was not statistically significant (2.3 vs. 7.5%, *P* = 0.348), which may be related to the small sample size.

This study is the first mNGS study of BALF in infants with pulmonary infection after CHS. Pathogens were identified by the DNA levels of microorganisms in BALF samples and their relative abundance levels. This method significantly improves the sensitivity of pathogenic detection and shortens the time to diagnose pathogens, which provides an important reference for clinical anti-infection practice. However, this study also has certain limitations. The sample size of this study was small, and the infants with CHD showed a diverse distribution. Moreover, mNGS cannot provide the drug susceptibility results of pathogens, so the antimicrobial regimen can only be adjusted empirically after receiving the results. Additionally, as a new pathogenic microorganism detection technology, mNGS is more expensive than traditional pathogenic microorganism detection technology ($500 vs. $30), and; thus it is difficult to perform multiple tests. Although mNGS has been widely accepted and used in critically ill infected patients, mNGS results should be combined with epidemiological and clinical features to identify causative microorganisms for technical reasons (e.g., methods to eliminate the influence of host genes). There is no uniform standard for the modification and guidance of clinical treatment strategies by mNGS results, especially for some refractory cases, which limits its application in clinical research. Finally, most of the infants in this study were given antimicrobial treatment before sampling, which may reduce the positive rate of CMT and the sensitivity of mNGS.

## Conclusion

The sensitivity of mNGS technology for the detection of the pathogen in severe pneumonia after CHS has been significantly improved. This can help to target and adjust antimicrobial drugs and improve the prognosis of infants. In the future, large sample, multicenter, prospective studies should be carried out to better understand the application of mNGS detection in CHD complicated with infection and improve the prognosis of children with CHD.

## Data availability statement

The original contributions presented in this study are included in the article/supplementary material, further inquiries can be directed to the corresponding author.

## Ethics statement

The studies involving human participants were reviewed and approved by the Fujian Maternity and Child Health Hospital. Written informed consent to participate in this study was provided by the participants or their legal guardian/next of kin.

## Author contributions

HC, QC, and Y-RZ designed the study, performed the statistical analysis, participated in the operation, and drafted the manuscript. Y-KC and S-HL collected the clinical data. All authors read and approved the final manuscript.
